# Unique Hepatic Arterial Pattern Associated With the Presence of Accessory Proper Hepatic Artery and Middle Hepatic Artery

**DOI:** 10.7759/cureus.65932

**Published:** 2024-08-01

**Authors:** Yashwanth Duddu, Alka V Bhingardeo, Srija Darna, Savithru Ganti, Mrudula Chandrupatla

**Affiliations:** 1 Anatomy, All India Institute of Medical Sciences, Bibinagar, Hyderabad, IND

**Keywords:** liver transplantation, accessory proper hepatic artery, coeliac trunk, middle hepatic artery, accessory hepatic artery, common hepatic artery, trifurcation

## Abstract

Vascular variations of the coeliac trunk are relatively common, with documented occurrences including trifurcation of the common hepatic artery (CHA) and the presence of accessory and replaced hepatic arteries. This case report describes a novel variation wherein the CHA trifurcates into the proper hepatic artery (PHA), gastroduodenal artery, and accessory PHA (APHA). This particular trifurcation pattern has not been previously recorded. The APHA further branches into two arteries that supply the right lobe of the liver. Additionally, a middle hepatic artery (MHA), originating from the PHA, was identified alongside the right and left hepatic arteries. The MHA serves as a hilar artery that drains segment IV of the liver. This anatomical variant does not conform to any existing coeliac trunk classifications. Understanding this unique arterial pattern is crucial for liver transplantation, as well as procedures involving the pancreas, duodenum, and gallbladder, and for interventional techniques such as transcatheter arterial chemoembolization and transarterial radionuclide therapy.

## Introduction

Three ventral branches of the abdominal aorta-the coeliac trunk, the superior mesenteric artery (SMA), and the inferior mesenteric artery-supply arterial blood to the gastrointestinal system [1,2]. The coeliac trunk, which arises from the abdominal aorta at the level of the upper part of the L1 vertebra [1], typically trifurcates into the splenic artery, common hepatic artery (CHA), and left gastric artery (LGA). The CHA further bifurcates into two terminal branches: the proper hepatic artery (PHA) and the gastroduodenal artery (GDA) [2]. The PHA then divides into the right and left hepatic arteries, which provide blood to the right and left lobes of the liver, respectively [1].

Hepatic arterial variations are prevalent [3], with Michels’ classification and its modification by Hiatt being the most commonly used system [3]. This case report presents a distinctive variant of the coeliac trunk, characterized by the trifurcation of the CHA into the GDA, PHA, and an accessory PHA (APHA). Notably, this specific trifurcation pattern involving the CHA has not been previously documented in the literature.

Anatomical variations of the hepatic arteries and coeliac trunk are crucial for liver and pancreatic transplantation, hepatic arteriography, radiological abdominal interventions, and various laparoscopic procedures [2]. A thorough understanding of these arterial variations is vital to prevent accidental injuries and hemorrhage during such procedures [4,5].

## Case presentation

**Figure 1:**
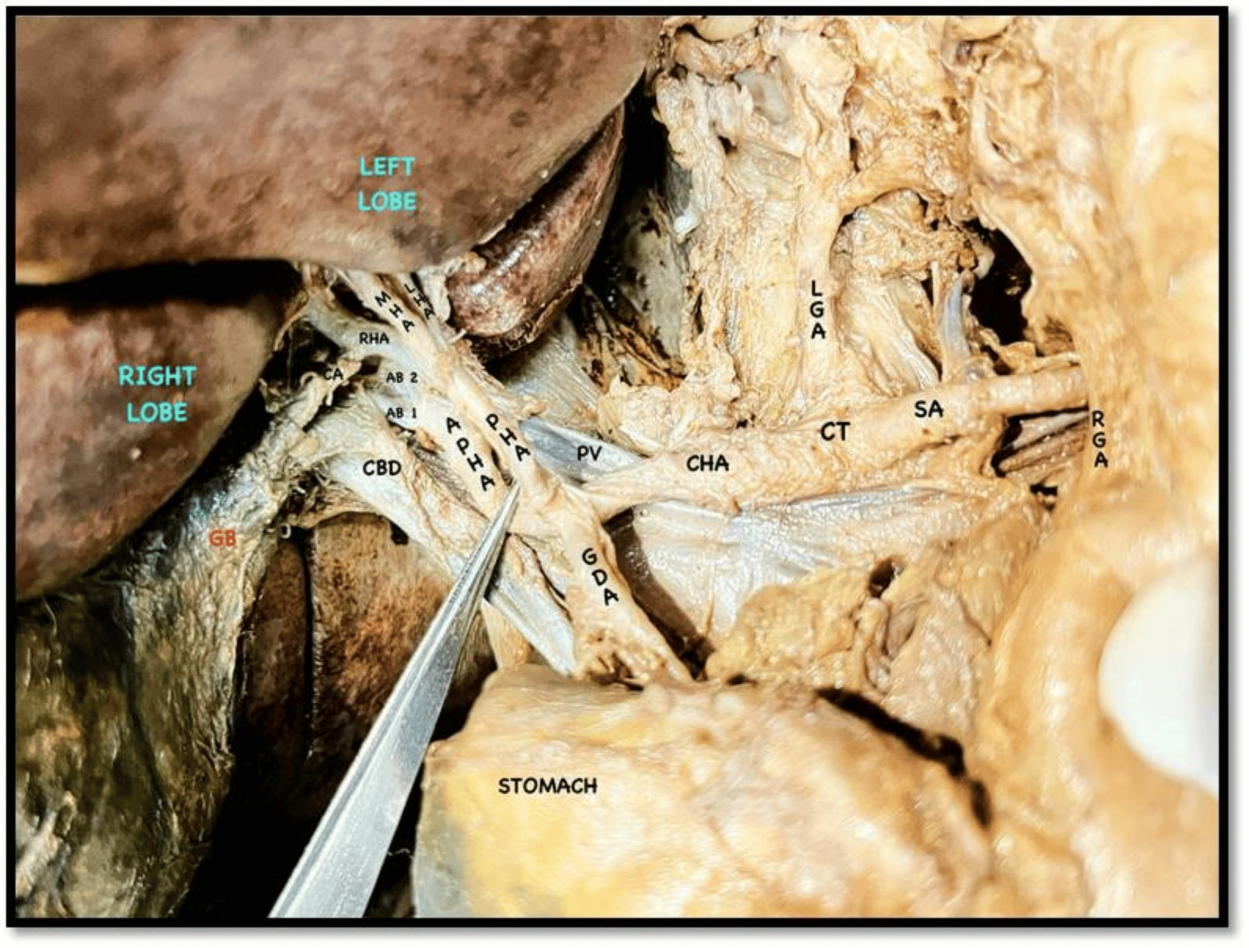
Coeliac trunk and its branches, APHA and its branches (AB1 and AB2), and PHA and its branches (RHA, MHA, and LHA) AB1, arterial branch 1; AB2, arterial branch 2; APHA, accessory proper hepatic artery; CA, cystic artery; CBD, common bile duct; CHA, common hepatic artery; CT, coeliac trunk; GB, gall bladder; GDA, gastroduodenal artery; LGA, left gastric artery; LHA, left hepatic artery; MHA, middle hepatic artery; PHA, proper hepatic artery; PV, portal vein; RGA, right gastric artery; RHA, right hepatic artery; SA, splenic artery

**Figure 2:**
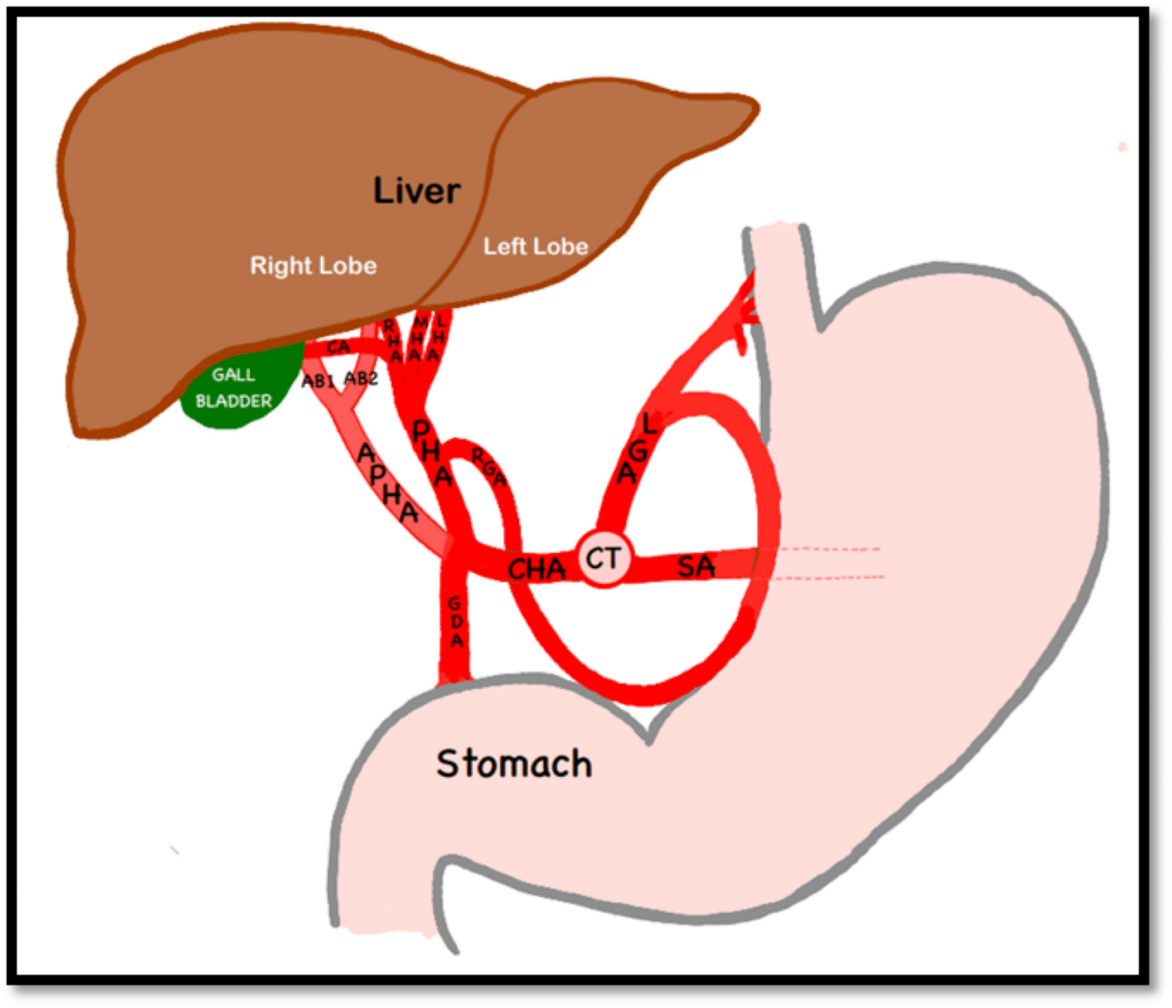
Schematic representation of the dissection image shown in Figure 1 (original work of the authors, for better understanding of readers) AB1, arterial branch 1; AB2, arterial branch 2; APHA, accessory proper hepatic artery; CA, cystic artery; CBD, common bile duct; CHA, common hepatic artery; CT, coeliac trunk; GB, gall bladder; GDA, gastroduodenal artery; LGA, left gastric artery; LHA, left hepatic artery; MHA, middle hepatic artery; PHA, proper hepatic artery; PV, portal vein; RGA, right gastric artery; RHA, right hepatic artery; SA, splenic artery

**Figure 3:**
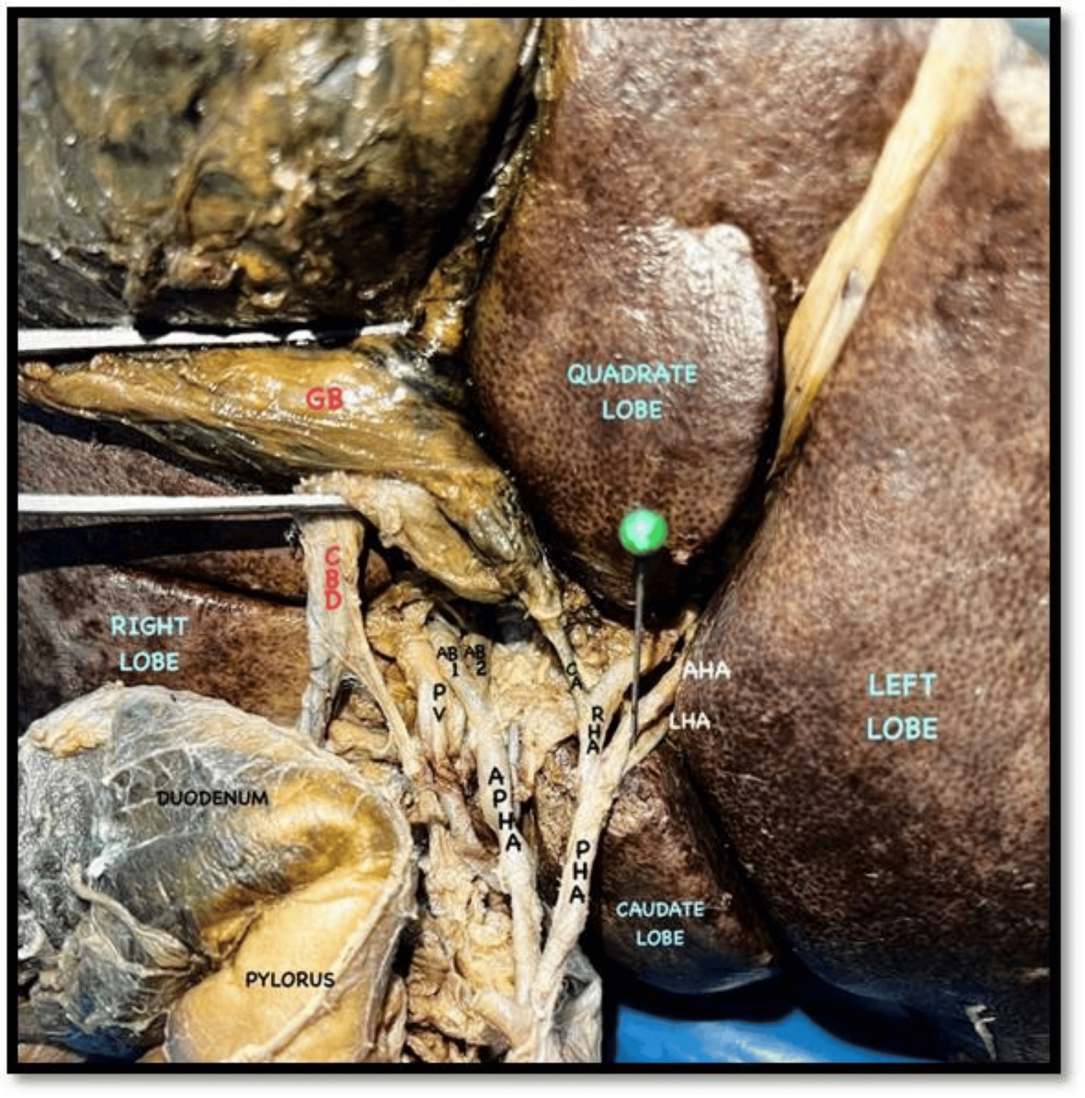
APHA and its branches (AB1 and AB2) and PHA and its branches (RHA, MHA, and LHA) (closer view) AB1, arterial branch 1; AB2, arterial branch 2; APHA, accessory proper hepatic artery; CA, cystic artery; CBD, common bile duct; CHA, common hepatic artery; CT, coeliac trunk; GB, gall bladder; GDA, gastroduodenal artery; LGA, left gastric artery; LHA, left hepatic artery; MHA, middle hepatic artery; PHA, proper hepatic artery; PV, portal vein; RGA, right gastric artery; RHA, right hepatic artery; SA, splenic artery

During the routine cadaveric dissection of a 62-year-old male cadaver, we observed variations in the arterial pattern of the coeliac trunk. The coeliac trunk was divided into three cardinal branches: the splenic artery, the LGA, and the CHA (Figures 1, 2). Typically, the CHA branches into the GDA and continues as the PHA. In this case, we found a trifurcation of the CHA. The CHA was further divided into the PHA, the GDA, and an additional branch accompanying the PHA. We designated this additional artery as an APHA because it arose from the CHA alongside the PHA and supplied the liver (Figure 3). This APHA originated from the CHA between the PHA and GDA, traveled posteriorly to the PHA, and then was divided into two terminal branches that supplied the right lobe of the liver. The course of the GDA was normal. Unexpectedly, we also found a trifurcation of the PHA. It was divided into the right hepatic artery (RHA), the left hepatic artery (LHA), and an accessory hepatic artery (AHA) (Figure 3). The AHA was located between the RHA and LHA, and it terminated by supplying segment IV of the liver. We designated this artery as the MHA. The cystic artery arose from the RHA. The rest of the branches of the coeliac trunk and their courses were normal.

## Discussion

Haller first described the normal and aberrant anatomy of the coeliac trunk in 1976 [6]. Subsequent classifications of the coeliac trunk were proposed by Lipshutz, Adachi, Morita, and Michels [6-8].

Morita classified the coeliac trunk into five types: (1) normal coeliac trunk; (2) hepatosplenic trunk; (3) gastrosplenic trunk; (4) hepatogastric trunk; and (5) absent coeliac trunk. According to this classification, the coeliac trunk in our cadaver was a normal coeliac trunk [7].

Sureka et al. found that 7.16% of cases had a CHA that was divided into three branches: the RHA, LHA, and GDA, rather than the typical two branches. They also identified a few cases (2.16%) where the CHA was divided into four branches in their study [8]. In these trifurcation cases, the CHA branches included the RHA, LHA, and GDA, but the PHA was absent. A similar case of trifurcation was reported by Badagabettu et al. [9]. However, this trifurcation was associated with the absence of the coeliac trunk. The trifurcation pattern was similar to that mentioned by Sureka et al. [8].

Nayak and Vasudeva [10] also describe a similar trifurcation of the CHA in another case report, albeit in association with a varied course and branching pattern of the hepatic arteries. Unlike in their previous case, the coeliac trunk was present.

The trifurcation patterns of the CHA mentioned in the literature differ from those of our present case, as they are mostly associated with the absence of the PHA. In our cadaver, we found a trifurcation of the CHA into the GDA, PHA, and APHA. This case is unique, as we did not find any similar trifurcation patterns mentioned in the literature.

As per Sureka et al. [8], these arterial variations are significant in interventional procedures such as transcatheter arterial chemoembolization, placement of infusion pumps, and transarterial radionuclide therapy. In the presence of variations like the trifurcation of the CHA or double PHA, preoperative consideration of the requirement for more than one catheter to achieve adequate tumor perfusion is necessary [11]. Zanon et al. note that clamping or ligation of the CHA in trifurcation cases can lead to gastric or duodenal hypoperfusion [11].

The presence of AHA has been reported in the literature. AHA can originate from any artery of the coeliac trunk other than the PHA and supply the liver in addition to the RHA and LHA [12]. Replaced hepatic arteries have also been mentioned. These arteries are branches from any artery of the coeliac trunk except the PHA and serve as the main arterial blood supply to the liver in the absence of the RHA and LHA arising from the PHA [13].

In the present case report, we also observed a unique hepatic arterial pattern. The PHA branched into three arteries instead of two and supplied the liver. These three arteries were the RHA, LHA, and AHA. This AHA was located between the RHA and LHA and supplied segment IV, so we designated it MHA.

In addition to this blood supply, the liver also received blood from APHA, which further branched into two arteries and supplied the right lobe of the liver. Our literature review yielded no mention of such an APHA.

Elsamaloty et al. also observed the presence of the MHA arising from the CHA [14,15]. Wang et al. [8,12] classified the MHA into five types depending on its origin (Table 1).

**Table 1:** Wang’s classification of MHA CHA, common hepatic artery; LHA, left hepatic artery; MHA, middle hepatic artery; RHA, right hepatic artery

Types	Description	
	Origin of MHA	Other associated variations
Type I	RHA	No associated variations
Type II	LHA	No associated variations
Type III	RHA	Replaced LHA
Type IV	LHA	Replaced RHA
Type V	CHA, hepatic artery proper	No associated variations

The present variation can be classified as having a type V MHA according to Wang’s classification, as the MHA arose from the PHA.

Sureka et al. observed a majority of cases with the MHA originating from the RHA, followed by the LHA, and a few cases with the MHA arising from the CHA [8]. Khatiwada et al. [16] identified the presence of the MHA in 76% of cases in their study. Of these, the majority (34%) had a type I pattern where the MHA originated from the RHA, while 24% showed a type II pattern where the MHA originated from the LHA in the normal hepatic arterial configuration. The prevalence of types III, IV, and V was 6%, 8%, and 4%, respectively.

Injury to an MHA-supplying segment IV can lead to a functional reduction of lobes and ischemic cholangiopathy in liver transplantation procedures [4]. Michels et al. and Healey et al. identified an equal number of cases with the origin of MHA in the RHA and LHA. Sureka et al., Khatiwada et al., Kishi et al., Kamel et al., Jin et al., and Wang et al. reported the highest proportions of cases with the MHA originating from the RHA [14]. Michels et al. [4,15] proposed 10 types of hepatic arterial patterns in their study (Table 2).

**Table 2:** Michels’ classification of hepatic arterial patterns AHA, accessory hepatic artery; CHA, common hepatic artery; LGA, left gastric artery; LHA, left hepatic artery; SMA, superior mesenteric artery; RHA, right hepatic artery

Types	Description
Type I	Normal hepatic arterial pattern
Type II	The LHA is replaced by the LGA or its branch
Type III	The RHA is replaced by the superior mesenteric or its branch
Type IV	Both right and left hepatic arteries replaced
Type V	Presence of an accessory LHA
Type VI	Presence of an accessory RHA
Type VII	Both accessory right and left hepatic arteries present
Type VIII	Either the right or LHA is replaced and associated with the presence of an AHA
Type IX	CHA originating from the SMA
Type X	CHA originating from the LGA

The present case did not fit into any of these types. Type III (replaced RHA) is the most common variant (3.7%), according to Michels et al. [15].

Hiatt et al. [5,17] modified Michels’ classification and proposed a new classification of six categories. The first four categories are the same as those in Michels’ classification. Types V and VI are related to the origin of the CHA. If the CHA originates from the SMA, it is classified as type V, similar to type IX in Michels’ classification. Type VI refers to the isolated origin of the CHA in the abdominal aorta. The hepatic arterial pattern observed in our case cannot be included in any of these categories.

Multiple theories explain the embryological basis of varied arterial patterns. Mugunthan et al. [18] explained in their study that during embryonic development, the 10th segmental artery gives rise to the coeliac axis. The 11th and 12th segmental arteries regress, while the 13th segmental artery gives rise to the SMA. Following normal development, the coeliac trunk gives rise to three cardinal branches: the splenic artery, the CHA, and the LGA. The CHA further continues as the PHA after giving rise to the gastroduodenal branch and dividing into the RHA and LHA. The persistence of different embryonic blood supply elements gives rise to varied hepatic vascular patterns [18].

Gordon et al. state that an anastomotic channel interconnects primitive ventral branches of the aorta. Variations of the coeliac axis occur due to regression or overgrowth of these channels [8,19]. According to Madhu and Harish, the liver is initially supplied by the LHA from the LGA, the MHA or CHA from the coeliac trunk, and the RHA from the SMA. As development progresses, only the CHA remains persistent and supplies the liver by dividing into the RHA and LHA, while the remaining arteries regress [5,20].

Preoperative evaluation of hepatic artery variations in both the donor and recipient in liver transplantation is essential. In cases of variations in the hepatic arterial pattern in the donor, hepatic arterial reconstruction is necessary to ensure adequate perfusion of the graft [16]. The AHA is more prone to ischemic damage compared to normal arteries. The accessory RHA can sometimes be mistaken for the cystic artery and can be injured intraoperatively [3]. A lack of awareness of these arterial variations may lead to intraoperative injuries and subsequent failure of the liver and pancreas [3].

## Conclusions

This case was unique due to the multiple observed variations, including the trifurcation of the CHA and the presence of both an APHA and MHA. Our literature review found no evidence of this combination of variants in a single case. Understanding such variations in the hepatic arterial pattern is crucial for preventing unintentional injury during hepatic, biliary, and transplantation procedures, which could otherwise lead to severe hemorrhage or hepatic infarction.
